# Ethanol Modulates Spontaneous Calcium Waves in Axonal Growth Cones *in Vitro*

**DOI:** 10.3390/brainsci3020615

**Published:** 2013-04-23

**Authors:** Tara A. Lindsley, Joseph E. Mazurkiewicz

**Affiliations:** Center for Neuropharmacology & Neuroscience, Albany Medical College (MC-136), 47 New Scotland Ave., Albany, NY 12208, USA; E-Mail: mazurkj@mail.amc.edu

**Keywords:** ethanol, calcium waves, axonal growth cone, hippocampal culture

## Abstract

In developing neurons the frequency of long duration, spontaneous, transient calcium (Ca^2+^) elevations localized to the growth cone, is inversely related to the rate of axon elongation and increases several fold when axons pause. Here we report that these spontaneous Ca^2+^ transients with slow kinetics, called Ca^2+^ waves, are modulated by conditions of ethanol exposure that alter axonal growth dynamics. Using time-series fluorescence calcium imaging we found that acute treatment of fetal rat hippocampal neurons with 43 or 87 mM ethanol at an early stage of development in culture decreased the percent of axon growth cones showing at least one Ca^2+^ wave during 10 min of recording, from 18% in controls to 5% in cultures exposed to ethanol. Chronic exposure to 43 mM ethanol also reduced the incidence of Ca^2+^ waves to 8%, but exposure to 87 mM ethanol increased their incidence to 31%. Neither chronic nor acute ethanol affected the peak amplitude, time to peak or total duration of Ca^2+^ waves. In some experiments, we determined the temporal correlation between Ca^2+^ waves and growth and non-growth phases of axonal growth dynamics. As expected, waves were most prevalent in stationary or retracting growth cones in all treatment groups, except in cultures exposed chronically to 87 mM ethanol. Thus, the relationship between growth cone Ca^2+^ waves and axon growth dynamics is disrupted by ethanol.

## 1. Introduction

Maternal consumption of alcohol during pregnancy can produce abnormalities in the central nervous system of the fetus, resulting in life-long cognitive and behavioral impairments called fetal alcohol spectrum disorder (FASD) [1]. Brain imaging studies in humans and experimental animals exposed prenatally to alcohol show disordered fiber tracts in the corpus callosum and other brain regions [2,3,4]. These, and other histopathologic evidence, are consistent with ethanol disruption of neuronal process outgrowth [5]. Calcium (Ca^2+^) signaling is a well-known regulator of axon growth and guidance [6,7,8] and some Ca^2+^ signaling components, such as Ca^2+^ channels, are sensitive to ethanol [9].

Spontaneous fluctuations in [Ca^2+^]_i_ in axonal growth cones regulate axon growth dynamics, which are characterized *in vitro* and *in vivo* by alternating periods of growth when length increases, and periods of non-growth when axons pause [10]. In previous studies, we found that chronic exposure to 43 or 87 mM ethanol in the medium of low-density cultures of embryonic rat hippocampal pyramidal neurons delays initial axon outgrowth and disrupts axon growth dynamics [11]. Once initiated, the rate of axon growth overall was increased by ethanol, primarily due to a decrease in retraction that occurred during non-growth periods. We also observed a small decrease in the duration of non-growth periods and a trend to increased rate of extension during growth periods, though these differences were not statistically significant. Further investigation showed that the same conditions of ethanol exposure that altered axon growth dynamics also inhibited depolarization-induced Ca^2+^ signaling and altered the function and expression of Ca^2+^ channels in axonal growth cones [12]. However, whether ethanol also affects spontaneous, growth-regulating Ca^2+^ transients in growth cones is unknown.

There are two classes of spontaneous Ca^2+^ transients that regulate axon growth, spikes and waves (reviewed in [13]). Spikes and waves can both be eliminated by removal of extracellular Ca^2+^. Spikes have fast kinetics, are depolarization-dependent and involve Ca^2+^ influx through voltage-gated calcium channels (VGCCs). In contrast, waves have slow kinetics, are independent of depolarization and do not act via VGCCs. Slow, spontaneous Ca^2+^ waves localized to the axonal growth cones of *Xenopus* spinal neurons have been observed *in vitro* [14,15] and *in vivo* [16]. Fast, spontaneous Ca^2+^ spikes in axonal growth cones of Syrian hamster cortical neurons *in vitro* regulate axon branching as well as axon growth [17]. In both types of neurons, the frequency of these Ca^2+^ transients is inversely correlated with the rate of axon elongation, and increased frequency is characteristic of stationary growth cones. 

In the present study, we used time-series Fluo-3 imaging of growth cone Ca^2+^ to assess effects of ethanol on slow, spontaneous Ca^2+^ waves in hippocampal pyramidal neurons under the same conditions that alter axon growth dynamics. We show for the first time that ethanol alters the incidence of these Ca^2+^ waves in axonal growth cones and disrupts their relationship to axon extension.

## 2. Results and Discussion

### 2.1. Spontaneous Ca^2+^ Waves Occur in Axonal Growth Cones of Developing Hippocampal Neurons

**Figure 1:**
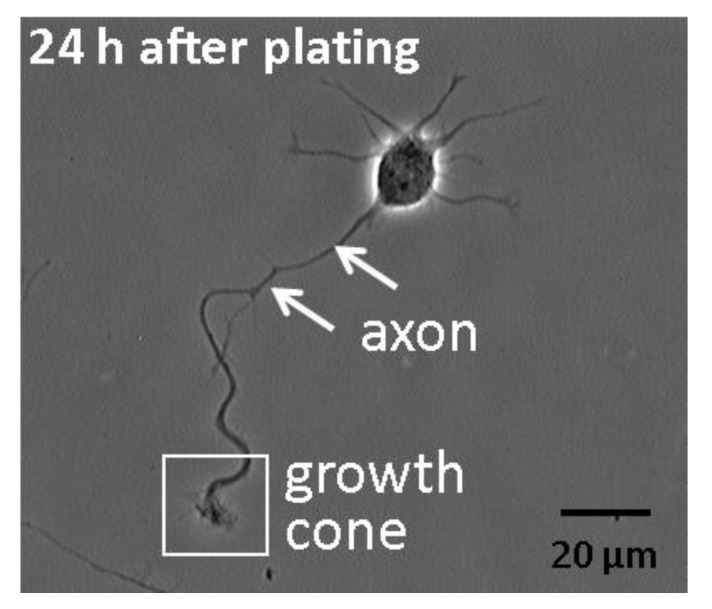
Morphology of hippocampal pyramidal neurons at an early stage of development in low-density dissociated cultures. Shown is a phase contrast image of a representative embryonic rat hippocampal pyramidal neuron 1-day after plating. It illustrates the typical appearance of the newly formed axon, tipped by a growth cone. Several shorter processes emerging from the cell body are nascent dendrites.

To determine whether Ca^2+^ waves occur in dissociated hippocampal neurons, we loaded fetal rat hippocampal neurons with the Ca^2+^ indicator dye Fluo-3 and imaged their axonal growth cones for 10 min. Imaging was performed on cultures 24 h after plating, when axons are rapidly extending but not intermingled with other processes. Only pyramidal neurons, which make up greater than 90% of the cells in these cultures, were imaged. Axons were identified by their length, which had to be longer than 40 µm and also longer than the next longest process by at least 20 µm [18] (Figure 1).

**Figure 2:**
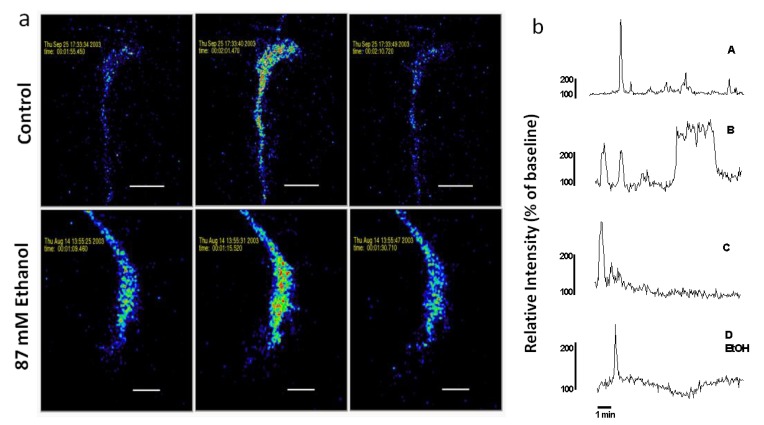
(**a**) Spontaneous Ca^2+^ waves in axonal growth cones of control and acute ethanol-exposed hippocampal pyramidal neurons. Time-series confocal images of cells loaded with Fluo-3 demonstrate a Ca^2+^ wave in a control neuron (top panels), and in a neuron exposed to 87 mM ethanol (bottom panels) added 15 min before starting the recording. Images are pseudocolored with peak elevations of [Ca^2+^]_i_ indicated in red. (**b**) Plots of relative fluorescence over time in representative growth cones illustrate variation in temporal pattern of waves. Traces A–C are typical Ca^2+^ waves recorded in the growth cones of neurons in control medium without ethanol. Trace D shows a Ca^2+^ wave observed when ethanol is added to the medium 15 min before imaging. Trace A is the growth cone on top in (**a**). Trace D EtOH is the growth cone on the bottom in (**a**).

Calcium waves were defined according to the criteria of Gomez *et al.* [15], which includes localization to the growth cone, fluorescence increases >150% of baseline, slow rise time (>3 s to peak), and return to baseline. Rarely, Ca^2+^ transients propagated to the growth cone from the cell body or axon shaft, and these were excluded from the analyses. These characteristics distinguish Ca^2+^ waves from Ca^2+^ spikes and from morphological movements and noise. To determine whether spontaneous Ca^2+^ waves also occur in neurons exposed to ethanol, ethanol was added to the medium (final concentration of 43 or 87 mM), either acutely or chronically. For acute exposure, ethanol was added 24 h after plating in normal medium, just before imaging. For chronic exposure, ethanol was added shortly after plating and was present in the medium continuously until imaging 24 h later. In both conditions, the concentration of ethanol in the medium was also present in buffers during imaging. None of these experimental conditions resulted in any observable effect on baseline calcium levels, neuron survival or growth cone morphology, either during 10 min recordings or 60 min afterwards. Calcium waves with varying temporal patterns were observed in axonal growth cones in all treatment groups. Figure 2 shows spontaneous Ca^2+^ waves in growth cones of control and ethanol-treated neurons.

### 2.2. Ethanol Alters the Incidence of Spontaneous Growth Cone Ca^2+^ Waves

The frequency of spontaneous Ca^2+^ waves in axonal growth cones in dissociated neuronal cultures varies depending on the cell type, but is generally low (1–8/h) under physiologic conditions. Since continuous imaging for more than a few minutes is associated with photobleaching, dye compartmentation and cell toxicity, we did not monitor the frequency of waves in the same growth cone. Instead, frequency of waves was determined indirectly by examining the mean incidence of axonal growth cones displaying at least 1 wave during a 10 min interval of recording, a method applied in other similar studies. For example, about 20% of chick dorsal root ganglion neurons at early stages of development in culture display at least 1 growth cone Ca^2+^ wave during a 10 min recording [15]. When localized waves in cell bodies and in growth cones are counted, about 38% of embryonic *Xenopus* spinal neurons display Ca^2+^ waves during 60 min period [19] and 50% of differentiating mouse striatal neurospheres show waves over 10 min [20]. The reliability of this method is supported by evidence from other studies showing that when the frequency of Ca^2+^ spike events is estimated by continuous imaging for 6 min, the calculated spikes/neuron × h yields estimates that are comparable to those obtained by measuring incidence over 10 min [19].

**Figure 3:**
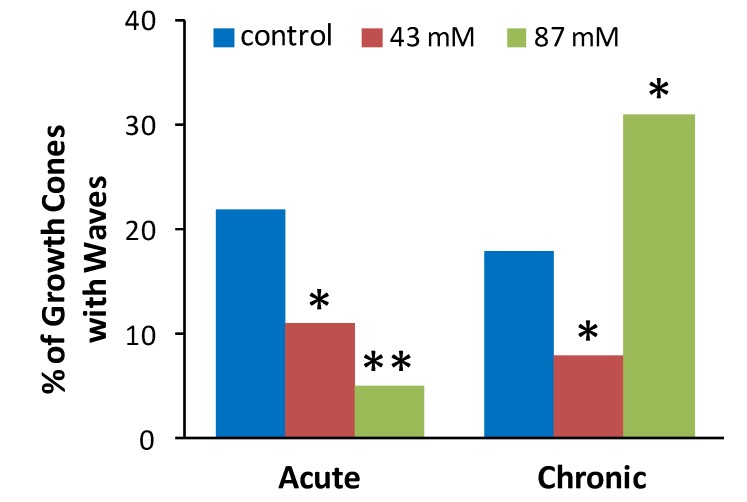
Ethanol alters the incidence of spontaneous calcium waves in axonal growth cones. The plot shows the percent of neurons in each treatment group that exhibited at least one Ca^2+^ wave during the 10 min. recording. The data are combined from 39 separate cultures with a total of 453 growth cones analyzed (54–91 per treatment group). * *p* < 0.05, ** *p* < 0.001, chi square, compared to control group for acute or chronic treatment.

We, therefore, determined the effect of acute or chronic ethanol exposure on incidence of spontaneous Ca^2+^ waves in axonal growth cones of hippocampal neurons in dissociated cultures Figure 3 shows the proportion (%) of axonal growth cones in each treatment group that displayed at least 1 wave during a 10 min recording period. Chi square tests were performed to determine if the observed proportion of growth cones displaying waves after ethanol treatment was different than the expected proportion as indicated by proportion of control growth cones displaying waves. Compared to control cultures without ethanol, the incidence of spontaneous waves was decreased by acute or chronic exposure to 43 mM ethanol and by acute exposure to 87 mM ethanol. In contrast, chronic exposure to 87 mM ethanol increased the incidence of growth cones with waves.

### 2.3. Ethanol Has No Effect on the Kinetics of Spontaneous Ca^2+^ Waves

**Figure 4:**
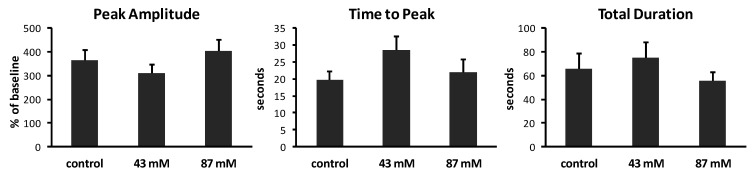
Characteristics of Ca^2+^ waves in axonal growth cones. Calcium imaging at 1 image/s generated plots of fluorescence over time. Analysis was performed on axonal growth cones that displayed at least one Ca^2+^ wave during 10 min (*n* = 14–15 growth cones per treatment group). Peak amplitudes of waves are expressed as the mean percent of baseline fluorescence (±SEM). Time to peak and total duration of waves are expressed as the mean time in seconds (s) (±SEM) to reach maximum fluorescence and to rise to the peak and return to baseline, respectively.

To determine whether chronic ethanol exposure alters the kinetic characteristics of spontaneous Ca^2+^ waves, we measured the peak amplitude, time to peak and total duration of waves in axonal growth cones from each treatment group. Results are summarized in Figure 4. In control cultures without ethanol, Ca^2+^ waves had varied amplitudes, ranging from 202% to 707% above baseline (mean = 363% ± 46.6%). The time to peak also varied, ranging from 3 to 40 s (mean = 19.8 ± 2.4 s). The total duration of waves ranged from 21.1 to 203 s (mean = 65.4 ± 13.2 s). The same analyses of Ca^2+^ waves in cultures containing ethanol in the medium, added shortly after plating, showed no significant effects on peak amplitude (*p* = 0.17; ANOVA), on time to peak (*p* = 0.20; ANOVA) or on total duration (*p* = 0.52; ANOVA).

### 2.4. Regulation of Axon Growth by Ca^2+^ Waves Is Altered by Ethanol

Others have reported that Ca^2+^ transients are most prevalent in growth cones when they pause between periods of active extension, and are rarely observed during growth phases when length increases [15,17]. To determine whether ethanol affects the relationship between phase of axon growth and Ca^2+^ dynamics, we captured a single image of axonal growth cones 50 min after a Ca^2+^ wave and recorded whether it had advanced at least 20 µm (growth period), or had remained stationary or retracted (non-growth period). Results are summarized in Table 1.

**Table 1:** The percent of wave-displaying growth cones which are in periods of non-growth.

Treatment	Number in non-growth period	Number in growth period	% in non-growth period
*Acute ethanol*			
Control (0 mM)	21	7	86%
43 mM	10	2	83%
87 mM	8	2	80%
*Chronic ethanol*			
Control (0 mM)	26	6	81%
43 mM	10	2	83%
87 mM	14	34	26% **

Acute ethanol: Neurons plated and maintained in medium without ethanol, then ethanol added just before imaging; Chronic ethanol: Ethanol added to medium shortly after plating, then present in buffers during dye loading and imaging; ** p < 0.0001; compared to control: chi square.

As expected, based on previous studies [15,17], in control cultures without ethanol in the medium more than 80% of the axonal growth cones that exhibited Ca^2+^ waves had not extended at least 20 μm when examined 50 min after the wave was recorded. These results indicate that the Ca^2+^ waves likely occurred during periods of non-growth. Although only a small proportion of axonal growth cones displayed Ca^2+^ waves after acute exposure to 43 or 87 mM ethanol (Figure 3 above), those that did were much more likely to be in a period of non-growth than in a period of growth when the wave occurred as evidenced by the length of axon extension when examined 50 min subsequent to the wave. Similar results were observed for growth cones that displayed Ca^2+^ waves after chronic exposure to 43 mM ethanol in the medium. Thus, for both acute alcohol at 43 and 87 mM and chronic ethanol at 43 mM, ethanol appears to have decreased the frequency of Ca^2+^ waves in axonal growth cones without affecting the correspondence between the expression of waves and non-growth periods that is normally observed. In surprising contrast, after chronic exposure to 87 mM ethanol, 34 out of 48 (74%) of axonal growth cones that displayed Ca^2+^ waves had extended at least 20 μm, indicating the wave occurred during a growth period. These results suggest that continuous exposure to ethanol at higher concentrations prevents the normal braking effect of Ca^2+^ waves on axon growth, even as it increases wave frequency. 

We report here that developing hippocampal neurons in dissociated cultures displayed spontaneous Ca^2+^ transients in their axonal growth cones. The low frequency, slow kinetics and localization of these transients are consistent with their identity as Ca^2+^ waves. Furthermore, these transients showed the inverse relationship to axon growth that is characteristic of waves. Specifically, axons in non-growth periods were more likely to display Ca^2+^ waves than axons in growth periods. Acute exposure to 43 or 87 mM ethanol decreased the frequency of Ca^2+^ waves in axonal growth cones, but did not affect their kinetics or their inverse relationship to axon growth. Continuous exposure to 43 mM ethanol in the medium had a similar effect. Unexpectedly, although continuous exposure to 87 mM ethanol also had no effect on the kinetics of waves, it greatly increased their frequency, but suppressed the inhibition of axon growth expected when the frequency of Ca^2+^ waves is high. Thus, both the frequency of Ca^2+^ waves and their regulation of axon growth dynamics were perturbed by ethanol in these cultures, albeit in a complex manner. 

We previously reported that chronic exposure to 43 or 87 mM ethanol in hippocampal pyramidal neuron cultures increases the overall growth rate of axons, by inhibiting retractions when axons pause [11]. We also noted that axons averaged 20% less time in non-growth periods than control neurons, though this difference was not statistically significant. The chronic exposure to 43 mM ethanol that enhanced overall rate of axon growth in our previous study decreased the proportion of growth cones with waves in the current study. This finding was not unexpected, given that the frequency of Ca^2+^ waves is normally decreased under conditions that increase rate of axon growth. It was surprising however, that chronic exposure to 87 mM ethanol which also enhanced overall rate of axon growth in the previous study *increased* the proportion of growth cones with waves in the current study. Normally such an increase in wave frequency would be expected to decrease the rate of axon growth, but our previous findings showed the effects ethanol on axon growth dynamics were similar at 43 and 87 mM. Taken together, it appears that the correlation between frequency of waves and axon pausing may be lost at the higher concentration of ethanol, perhaps because ethanol interferes with other cellular functions or extracellular conditions that favor axon growth in this culture system.

We also recently reported that acute and chronic exposure to ethanol at the same concentrations used in the present studies inhibits the amplitude of KCl-induced Ca^2+^ transients in axonal growth cones [12]. This was true despite up-regulation of L-type channel expression that occurs with prolonged exposure to ethanol. Like KCl-induced calcium spikes, spontaneous calcium waves require Ca^2+^ influx, as they can be blocked by Ni^2+^ and disappear when extracellular Ca^2+^ is removed, but waves do not involve Ca^2+^ influx through VGCCs because they are not affected by agents that block these channels [19]. Instead, Ca^2+^ entry associated with waves has been proposed to be through an unidentified channel activated perhaps by mechanical forces at the growth cone, by products of local metabolism or by depletion of Ca^2+^ stores [13]. Although we did not assess ethanol effects on Ca^2+^ release from intracellular stores in the current study, this seems unlikely to contribute to waves because wave amplitude was not affected by ethanol under any treatment conditions. 

What can be learned from ethanol disruption of Ca^2+^ wave frequency? Although regulation of axon growth dynamics is encoded by the frequency of waves [14], the underlying mechanism controlling cell-specific frequency is mysterious. Some evidence in developing *Xenopus* spinal cord indicates that neurons have an endogenous frequency of Ca^2+^ waves in axon growth cones that can be modulated up or down by changes either in extracellular cues or in the expression of axonal molecules that interact with those cues [16]. This raises the possibility that ethanol alters wave frequency by modifying an adhesive property of the extracellular matrix bound to the culture substrate, or an axonal protein that binds to that matrix. What can be learned from ethanol disruption of wave-regulated axon growth? In *Xenopus* spinal neurons the mechanism underlying control of axon growth by spontaneous Ca^2+^ waves requires calcineurin (CN), a Ca^2+^-dependent protein phosphatase, which acts downstream of waves [21]. Pharmacological treatments that inhibit CN or stabilize actin filaments can prevent the growth-inhibiting effect of Ca^2+^ waves, much the way ethanol did in the current study. This raises the possibility that prolonged high concentrations of ethanol may act similarly in mammalian neurons by directly inhibiting CN or other components of the signaling cascade downstream of waves. Interestingly, some substrates of CN are sensitive to ethanol, such as GAP-43 which is down-regulated and dephosphorylated in rat hippocampus by short-term ethanol treatment [22]. In the future, it will be important to relate our findings in dissociated cultures to ethanol effects on growth-regulating spontaneous Ca^2+^ waves *in vivo*, where the complex cellular and molecular environment of the developing hippocampus is preserved.

## 3. Experimental Section

### 3.1. Culture of Primary Embryonic Hippocampal Neurons

Low-density cultures of primary hippocampal neuron cultures were prepared from the hippocampi of fetal Sprague-Dawley rats (Taconic Farms) at gestational day 19, as described by Kaech and Banker [23]. Briefly, we dissected the hippocampi from the cerebral hemispheres, removed the meninges, collected them in HEPES-buffered salt solution, dissociated the cells with trypsin (0.25% for 15 min at 37 °C) and triturated with a fire-polished Pasteur pipette. We plated the cells in Minimal Essential Medium (MEM) with 10% heat-inactivated horse serum at a density of 5650 cells/cm^2^ onto acid-etched glass coverslips precoated with poly-d-lysine. The plating medium was replaced 2 h later with cN2.1 medium (MEM with N2 supplements, 0.1% ovalbumin, 0.1 mM pyruvate, and 10 mM HEPES, conditioned by exposure to confluent cortical astrocytes for 2 days). In some dishes, 100% USP ethanol was added to the cN2.1 medium to achieve a final concentration of 43 or 87 mM, just prior to its transfer to the neurons, and remained in the medium continuously (chronic ethanol exposure). These concentrations of ethanol are comparable to maternal blood levels of 200 to 400 mg/dL, which is correlated with increased risk of CNS damage to the fetus [24]. Control and ethanol-treated cultures were maintained in modular incubator chambers at 36 °C in 5% CO_2_ saturated with water or water/ethanol at the target concentration in the medium, to prevent evaporation of ethanol from the medium [25]. More than 90% of the cells in these cultures are pyramidal neurons that are easily distinguished from GABAergic interneurons and non-neuronal cells based on morphological criteria, as described previously [25,26]. Axons were identified by their length measured using ImageJ software (NIH, Bethesda, MD, USA), which had to be longer than 40 µm and also longer than the next longest process by at least 20 µm [18].

### 3.2. Time-Lapse Ca^2+^ Imaging of Axonal Growth Cones

Twenty to 24 h after plating, we performed time-series Ca^2+^ imaging, essentially as described by Shitaka *et al.* [27], but using a Noran Oz Confocal Laser Scanning system to image Fluo-3 calcium at a high rate of capture (1 image/1 s). Hippocampal neurons were loaded for 30 min at room temperature with Fluo-3/AM (Invitrogen; 5 mM in DMSO diluted to a final incubation concentration of 2 μM) in Krebs-Ringer solution (150 mM NaCI, 5 mM KCI, 1 mM MgSO_4_, 2 mM CaCl_2_, 10 mM glucose, 10 mM HEPES, pH 7.4). After loading, the cultures were washed and incubated in Krebs-Ringer for 30 min to allow hydrolysis of the ester. For neurons that were exposed to ethanol continuously since plating, the same concentration of ethanol was present in solution for Fluo-3 loading and washing to prevent sudden withdrawal from ethanol. In some dishes containing neurons maintained in normal medium, 100% USP ethanol was added to the Krebs-Ringer just before imaging (acute ethanol exposure) to achieve a final concentration of 43 or 87 mM. Under these conditions, bright fluorescence emission was detected in all cells examined. After loading, coverslips were transferred to a Warner Instruments series 20 perfusion chamber and placed on the stage of a Nikon diaphot 200 inverted microscope coupled to a Noran Oz Confocal Laser Scanning System. For image acquisition of axonal growth cones we collected 1.3 frames/sec (jump averaging 4×) using a 100× 1.4NA oil lens, zoomed up (2–4×) to focus on individual growth cones over a 10 min time series. In some experiments, one additional image was captured 50 min after the end of the time series to assess neuron survival and to assess phase of growth (see below). Laser intensity was set at 4%–6%. Background fluorescence for each image stack was based on a cell-free portion of the culture substrate. Changes over time in relative intensity within a user-defined region positioned at the center of axonal growth cones were scored as a Ca^2+^ wave if the rise to peak was ≥3 s, the peak amplitude was ≥150% above baseline, and it returned to baseline, as defined by Gomez *et al.* [15]. The peak amplitude was the intensity at peak fluorescence before return to baseline, expressed as a percent of baseline. The rise time of a Ca^2+^ wave was measured as the interval between the baseline at the initiation of the wave and peak fluorescence intensity. The total duration was the time between initiation of the wave and return to baseline. 

### 3.3. Analyses Relating Observance of Ca^2+^ Waves and Phase of Growth

To assess the temporal correspondence of Ca^2+^ waves with axon growth dynamics, we used a Noran-movie plugin to export the image stack to ImageJ, and measured the distance between the leading edge of the growth cones in the last image of the 10 min time series and in the image taken 50 min later. Growth cones that extended at least 20 µm in the direction of the leading edge were considered to be in a growth period, and all others were considered to be in a non-growth period. These criteria were selected based on our previous finding that the rate of axon elongation during a growth period in these cultures is approximately 20 µm/h, that during periods of non-growth axons typically retract between 3.0 and 7.0 µm/h and that axons typically spend 1–3 h in one phase before transitioning into the other [11,28]. These threshold values allowed us to identify sustained growth and non-growth periods with reasonable certainty, without subjecting the growth cone to repeated imaging that reduced survival.

## 4. Conclusions

Here we report that exposure to ethanol in the medium of embryonic rat hippocampal pyramidal neurons during early development in culture alters the frequency and growth-regulating properties of spontaneous Ca^2+^ waves localized to the axonal growth cone. Our findings corroborate numerous reports documenting ethanol effects on Ca^2+^ signaling in neurons, in which Ca^2+^ channels and receptors figure importantly in cellular adaptation to ethanol and withdrawal-induced neuronal damage [9,29], but expands the list of ethanol sensitive signaling to include spontaneous Ca^2+^ oscillations in growth cones of developing neurons. Specifically, we found that ethanol can alter these growth-modulating events at concentrations comparable to blood levels associated with increased risk of neurologic damage to the human fetus [24]. Given that spontaneous Ca^2+^ transients are associated with pathways downstream of extracellular growth and guidance cues [8,13], our findings have potential implications for understanding how prenatal ethanol exposure disrupts neuronal process outgrowth and axon guidance, resulting in abnormal neuronal morphology and connectivity that characterizes FASD. Recent advances in live-imaging microscopy in intact mammalian brain [30] make it feasible for future studies to explore effects of ethanol on spontaneous Ca^2+^ waves in more native environments. 
